# Oncomeric Profiles of microRNAs as New Therapeutic Targets for Treatment of Ewing’s Sarcoma: A Composite Review

**DOI:** 10.3390/genes14101849

**Published:** 2023-09-23

**Authors:** Mubashir Hassan, Saba Shahzadi, Amal Malik, Salah ud Din, Muhammad Yasir, Wanjoo Chun, Andrzej Kloczkowski

**Affiliations:** 1 The Steve and Cindy Rasmussen Institute for Genomic Medicine, Nationwide Children Hospital, Columbus, OH 43205, USA; saba.shahzadi@nationwidechildrens.org; 2 Institute of Molecular Biology and Biotechnology, The University of Lahore, Lahore 54590, Pakistan; amalmalik.96@gmail.com; 3 Department of Bioinformatics, University of Okara, Okara 56130, Pakistan; salahuddin.gcuf@gmail.com; 4 Department of Pharmacology, College of Medicine, Kangwon National University, Chuncheon 24341, Republic of Korea; yasir.khokhar1999@gmail.com (M.Y.); wchun@kangwon.ac.kr (W.C.); 5 Department of Pediatrics, The Ohio State University, Columbus, OH 43205, USA

**Keywords:** Ewing’s sarcoma, miRNA, EWSR1, therapeutic targets

## Abstract

Ewing’s sarcoma is a rare type of cancer that forms in bones and soft tissues in the body, affecting mostly children and young adults. Current treatments for ES are limited to chemotherapy and/or radiation, followed by surgery. Recently, microRNAs have shown favourable results as latent diagnostic and prognostic biomarkers in various cancers. Furthermore, microRNAs have shown to be a good therapeutic agent due to their involvement in the dysregulation of various molecular pathways linked to tumour progression, invasion, angiogenesis, and metastasis. In this review, comprehensive data mining was employed to explore various microRNAs that might have therapeutic potential as target molecules in the treatment of ES.

## 1. Introduction

Ewing’s sarcoma (ES) is a rare, aggressive cancer, which particularly affects bones and soft tissues surrounding the bones [1,2,3]. ES affects any bone in the body, however, about 45% of cases have been located in the lower extremities, with another 45% in the pelvis, and 13% in the upper extremities, as well as the axial skeleton and ribs, and a further 2% in the face [4]. ES is an uncommon bone tumor that has a poor prognosis and a significant chance of spreading to the bone marrow and lung as its main sites of metastasis. ES tumors can be classified into askin tumors and peripheral primitive neuroectodermal tumors (pPNETs) based on the location of tumors in the human body. ES is considered the second death-causing bone tumour after osteosarcoma that affects children, adults, and young (less than 16 years old), respectively [5]. ES is controlled by the chromosomal translocation t(11;22) (q24;q12), which produces the fused EWSR1-FLI1 protein, a well-known transcription factor that regulates the activity of oncogenes.

The pathogenesis of ES remains unclear and there are cellular environmental factors that may possibly have an influence on the overexpression of the EWS-FLI1 complex. However, data show that this overexpression of EWS-FLI1 in primary cells causes an arrest in growth or even cell death. The differentiation defects in ES result in the enhanced expression of EWS/FLI in primitive cells that lack definitive lineage markers [6]. Another study reported that the formation of the EWS-FLI1 protein is responsible for biological alterations that cause neoplastic transformation in mesenchymal stem cells (MSCs) leading to the existence of ES [7]. However, further investigation is required to better understand the formation of the EWS-FLI1 gene and its involvement in ES. However, multiple published research reports showed that the EWS-FLI1 gene works as a transcriptional repressor and controls cellular proliferation and survival activities [6,8]. EWS-FLI1 also works as a transcriptional activator and promotes the expression of SRY-Box Transcription Factor 2 (SOX2), Insuline-like Growth Factor (IGF1), Enhancer of Zeste Homolog 2 (EZH2) genes respectively [9,10].

Around 22 percent of ES patients have metastases, and 14% have free survival rates. Additionally, the survival percentage for 5-year-old patients with no apparent metastases at diagnosis is 61% [11]. In the last few years, researchers have been trying to investigate some novel targets including genes, proteins, and microRNAs (miRs) to better understand the basic mechanism of ES [12,13,14,15]. Therefore, by knowing the significance of miRs in ES, the present review explores the miRs that could be used as therapeutic targets for ES and could be used in the designing of new chemical scaffolds and vaccines for the medication of ES.

## 2. Biosynthesis of miRs and Their Involvement in Cancers

miRs are small, composed of approximately 22 nucleotides, non-coding RNAs involved in switching on and off specific genes through transcriptional gene silencing (TGS) and changing their expression by post-transcriptional gene silencing (PTGS). miRs are accountable for the physiological and pathological processes related to cancer [16]. miRs are highly conserved and are transcribed by RNA polymerases II and III, respectively, which are responsible for the generation of the precursors that are then further subjected to a series of cleavages, which are nuclear and cytoplasmic in their nature. Additionally, miRs regulate at least 30% of the protein-coding genes and makeup roughly 1% to 5% of the human genome. The biogenesis of miR is a multi-step process that includes a nuclear and cytoplasmic phase as well as the participation of the ribonuclease III enzymes, dicer protein in the cytoplasm, and drosha protein in the nucleus.

**Figure 1:**
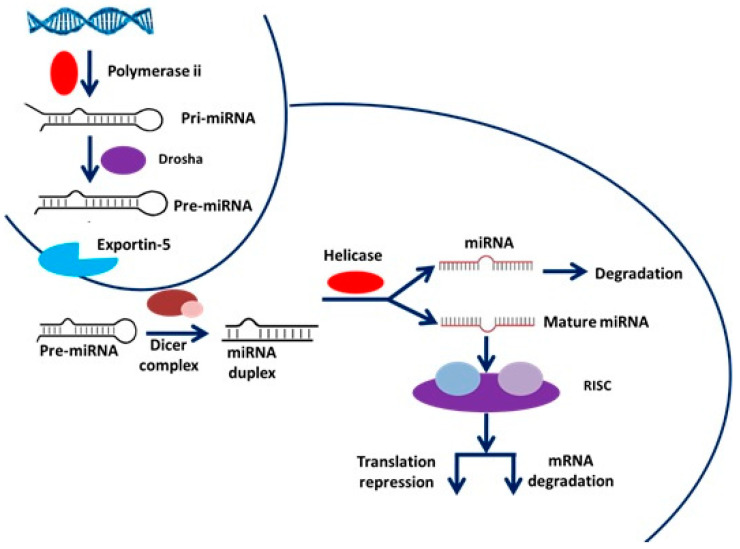
The basic mechanism of miR biogenesis [22].

The miR transcripts are first formed by RNA polymerase II and then go through a processing step, allowing miR synthesis according to specific conditions and cell types. Multiple research studies have reported that miRs are significantly implicated in stem cell pluripotency and somatic cell reprogramming [17,18,19,20]. Genes for miRs are transcribed into primary miRs, and handed to the drosha enzyme in the nucleus, where they are processed. In this processing step catalysed by Drosha, the hairpin loop of the pre-miR is cleaved at the stem and removed [21] (Figure 1).

Numerous published data have shown the significant role of miRs in various diseases such as cancer, Alzheimer‘s, cardiovascular, and skeletal muscle hypertrophy [23,24,25,26]. The prior reports showed that miRs control the expression of various genes by binding with messenger RNA (mRNA) in the cell [27]. Such bound mRNA stops coding for a specific protein and is destroyed [28]. A miR associated with cancer is called oncomir (or oncomiR). Additionally, many studies described the functions of miRs in cancer biology as biomarkers and new therapeutic targets against cancer [29,30,31].

The deletion of the miR-15/-16 cluster is associated with increased expression of a leukemia-related anti-apoptotic target gene [32]. Moreover, different studies reported that miRs are linked with tumour biologies such as angiogenesis, metastasis, and cell death [33,34]. There are multiple reports which explore the functionality of miRs in promoting cell invasion and metastasis [35,36] along with tumour suppressors by inhibiting Epithelial Mesenchymal Transition (EMT) and metastasis [37,38]. Furthermore, it has also been found that miRs are involved in Cancer Stem Cells (CSCs) biology by targeting various transcription factors such as Oct4, Sox2, Lin28A, and KLF4, respectively [39,40,41,42]. Recent studies have shown that miRs are associated with chemoresistance and chemosensitivity in various cancers. Another study found that the overexpression of miR-105/93-3p activates the Wnt/β-catenin signalling pathway by activating SFRP1, leading to a promotion of chemoresistance in breast cancer [43].

## 3. miRs in ES Development and Progression

There are multiple studies suggesting a possible association of miRs with ES. The functional study of miR-30a showed a functional correlation between EWS-FLI1 and CD99 membrane glycoprotein which are two significant biomarkers and therapeutic targets for ES [44]. Moreover, there is also a report that miR-20b, miR-130b, and miR-34b act as oncomiRs (miRs associated with cancer) in ES progression [9]. According to Kawano et al. (2017), the modulation of Transforming Growth Factor β Receptor II (TGFBR2) by miRs contributes significantly to the advancement of ES. They observed an antagonistic association between the downregulation of TGFBR2 and the overexpression of miR-20b in humans. This clearly indicates the oncomiRic character of miR-20b and its potential to regulate the TGFRB2 pathway [45].

miR-130b has a direct association with ES metastasis and found that its overexpression leads to enhanced cellular invasion and migration ability [46]. Additionally, the molecular evidence demonstrates that miR-130b promotes the Cell Division Cycle 42 (CDC42) pathway by suppressing Rho GTPase Activating Protein 1 (ARHGAP1), which results in the constitutive activation of CDC42. The activation of P21 (RAC1) Activated Kinase 1 (PAK1), which positively targets gene expression and is responsible for sarcoma invasion and metastasis and creates a positive feedback loop by promoting miR-130b expression, is furthermore caused by the knockdown of ARHGAP1 gene expression and prolonged activation of CDC42 [46]. miR-34b is also involved in promoting proliferation, migration, and invasion of Ewing’s sarcoma cells by decreasing the levels of mRNA and Notch homolog protein 1 (Notch 1), respectively [47]. The prior data indicated that the Notch signalling pathway can act as an oncogene in human cancers [48]. Another miR linked with ES is miR-708-5p through a differential expression pattern. miR-708-5p is downregulated in ES cells and its overexpression is correlated with reduced proliferation of various tumours [49,50]. The overexpression of miR-708-5p suppresses the tumor invasiveness and migration, thanks to positively targeting Rho-GTPase Rho C effectors (ROCK1 and ROCK2) and Matrix Metallopeptidase 12 (MMP-12), respectively [51]. On the contrary, another oncomiR miR-181c acts as an ES tumor promotor by negatively targeting the Tumour Necrosis Factor Receptor Super Family Member 6 (TNFRSF6) in ES cells [52].

One prior report showed that both miR-193b and miR-124 can act as tumour suppressors and specifically, miR-193b has been shown its associated with tumorigenesis and inhibit tumour growth and metastasis [53]. The functional data reported that miR-124 target Zinc Finger Protein SNAI2 (SLUG), which regulate cellular motility, inducing epithelial to mesenchymal transition (EMT) and overexpression of the cell cycle Cyclin D2 regulator (encoded by the CCND2 gene). Moreover, miR-124 overexpression inhibits cell motility and block the cell cycle progression [54]. The miR-193 negatively correlated with receptor tyrosine-protein kinase erbB-4 (ErbB4 gene), which has been identified as a metastasis promoter in ES [55,56]. Similarly, miR-30d is associated with growth, malignant progression, and apoptosis in ES. The miR-30d is also considered a tumor suppressor and its overexpression is linked with poor prognosis in prostate cancer [57,58]. The mechanistic study also investigated how downregulating the production of matrix metalloproteinases-9 and -2 (MMP-9/-2) in ES cell lines led to an increase in miR-30d in those cells.

Additionally, miR-30d overexpression results in the accumulation of apoptotic cells, which increases the expression of the Bcl-2-like protein 4 (Bax) and concurrently decreases the levels of the B-cell lymphoma 2 (Bcl-2) proteins. Furthermore, the MEK/ERK and PI3K/Akt signalling pathways, which are involved in cell survival, proliferation, cell cycle progression, metabolism, and protein expression in ES cells, have been connected to miR-30d and other key signalling pathways in the evolution of tumours [59,60]. The functional study reported that miR-107 overexpression is correlated with cell cycle progression, apoptosis, and restrained cell cycle arrest. The molecular mechanism behind these effects is probably mediated by the negative target of miR-107 on the Hypoxia-Inducible factor-1β (HIF-1β) gene [61]. Another miR-185 is also known as oncomiR due to its low expression in ES tissues and cells and tumour suppressor activity in various cancers [62,63]. The prior study explores the role of miR-185 in ES progression by its overexpression in ES cell lines which reduces cell proliferation and colony formation ability through Bcl-2 and the E2F Transcription Factor 6 (E2F6) targets [64].

In ES, miRs such as miR-683 and miR-107 prevent tumor progression and cell proliferation. The ES cells demonstrated that miR-638 is downregulated, and its overexpression inhibits vascular endothelial growth factor A (VEGFA) function by suppressing mRNA and VEGFA protein expression. It also induces cell death and inhibits cell proliferation. While miR-107 appears to suppress angiogenesis by inhibiting the development of new blood vessels [65]. In another bioinformatic study, it has been reported that miR-21, upon expression in ES cells, works as a tumour suppressor by targeting and downregulating the expression of Activated Leucocyte Cell Adhesion Molecule (ALCAM) [66]. Whereas the downregulation of miR-199b-5p has been reported in ES cell lines in comparison with human MSCs (Table 1) [67].

The miR-181c is linked with cancer development and manifested as an oncogene in ES [72]. The miR-214-3p is exhibited oncomiR behaviour and is constitutively suppressed in cell lines. However, when the EWS-FLI1 and/or CD99 genes are silenced, it is once again expressed, and miR-214-3p function as a marker of tumour aggressiveness by restricting ES cell proliferation and migration and suppressing the expression of its target HMGA1 [73].

miRs have altered the expression levels in cancer and this changed miRs expression profiles have been identified in several malignancies, particularly in osteosarcoma and EWS, respectively. It has been studied that EWS/FLI1 makes changes in miR-145 and let-7a expression profiles in EWS. When EWS-FLI1 is knocked down, the miR-145 expression increases rapidly. It is markedly decreased in EWS cell lines [74]. In contrast, miR-145 transfection raised EWS-FLI1 levels while ectopic expression of miR-145 in Ewing’s sarcoma cell lines significantly decreased EWS-FLI1 protein. Like let-7a, HMGA2 is a direct EWS-FLI-1 target implicated in the tumorigenicity of EFT cells because of its impact on the target oncogene’s expression. A signature of five miRs (miR-34a, miR-23a, miR-92a, miR-490-3p, and miR-130b) was found to be an independent predictor of risk for disease progression and survival after microarray analysis of data from 49 individuals with primary ES [75].

## 4. The Functional Role of Let-7 Family in ES

**Figure 2:**
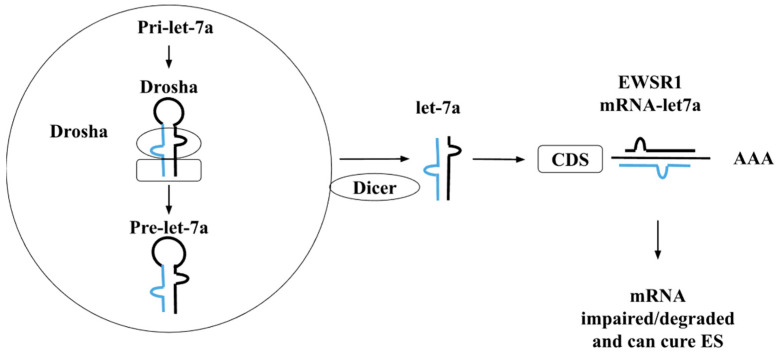
Mechanistic pathway of let-7a in ES. The blue and black color represents the 5’cap and poly A tail of miRNAs and after unwinding it binds with mRNA.

The members of the let-7 family, commonly known as tumour-suppressing miRs, have been involved in the downregulation of various kinds of cancers in comparison to normal, healthy tissues. There are a total of ten members of the let-7 family of miRs discovered in the human species, namely: let-7a, let-7b, let-7c, let-7d, let-7e, let-7f, let-7g, let-7i, miR-98, and miR-202, produced from approximately thirteen precursor sequences. Boyerinas et al. showed that a decrease in the let-7 family may lead to lower and poor survival rates of cancer patients [76]. The size of the family members of miRs varies, there are three basic separate precursor sequences of miRs such as let-7a-1, let-7a-2, and let-7a-3 for the production of let-7a. Similarly, let-7f is produced from two precursor miRs let-7f-1 and let-7f-2, respectively. It has been reported that five different miRs: let-7a, let-7b, let-7e, let-7f, and let-7g are directly associated with ES. Additionally, these miRs are linked with ES through the deregulation of miRs in 90% of ES patients. Furthermore, these observations were made through target gene prediction conducted of the deregulated miRNAs. A major correlation has been found through the observation of expression of let-7a and let-7b, especially a low expression of let-7b correlates with a higher risk of relapse of the disease [76]. Let-7 is known to have a crucial role in the negative regulation of oncogenic mutations of rat sarcoma virus (RAS), as well as in down or negative regulation of HIF-1α and EWS/FLI1 that are known to have an important role in the prognosis of ES. Therefore, let-7a has been reported as a direct target of EWS/FLI1 activity. The let-7a precursor sequence itself contains two mature miR sequences, 3p (containing 21 nucleotides) and 5p (containing 22 nucleotides), respectively. Another study showed that there is a link between the let-7a by EWS/FLI-1 [77]. The general mechanism through which let-7a expression controls the growth of ES is through the High Mobility Group AT-Hook 2 (HMGA2) protein-coding gene. As let-7a is overexpressed and HMGA2 in turn is repressed, both may work to block ES tumor cell tumorigenicity. Hence, there can be therapeutic use of overexpression and use of let-7a against the aggressive malignant tumour-forming mRNA and proteins, such as those in ES. The mechanistic study showed that let-7a is a direct EWS/FLI-1 target that has been evidenced to be involved in ES family tumors, this mechanistic pathway may be suggested as a possible (Figure 2). Firstly, the primary miRNA interacts with the Drosha enzyme processing complex and is converted into the precursor let-7a sequence. The Dicer enzyme further processes this precursor sequence into the mature sequence of let-7a. This mature sequence in turn may form a complex with the EWSR1 mRNA to silence this gene and disrupt the formation of the EWS protein, thus also impairing the formation of the EWS/FLI1 complex that occurs in ES [69,77]. The let-7 miRs having associations with carcinogenesis have been tabulated in Table 2.

## 5. miRs as Therapeutic Agents and Applications

Due to their ability to target multiple genes and cellular pathways altered in pathological conditions, miRs could be good candidates for novel therapeutic agents. miRs are involved in various diseases and play important roles due to their involvement in mutations and downregulations of key enzymes. The dysregulation of the miR biogenesis pathway has been linked in several studies to the emergence of cancer. Additionally, it has been noted that a poor prognosis is associated with the downregulation of the drosha and dicer enzymes, which has been found in numerous cancers [87,88].

ES is a paediatric sarcoma that usually originates in bones. Genetically, it arises from chromosomal translocations that fuse the EWS protein to different transcription factor members of the ETS family, with EWS-ETS fusion genes acting as the pathogenic catalyst for ES [12,89]. Additionally, some miRs could potentially target insulin-like growth factor (IGF) which is a known player for ES [12]. Another study identified miR-145 as a top miR candidate that is repressed in ES [89]. The EWS/FLI1 fusion transcript may be directly targeted by miR-145, which suggests that this regulatory network should be further investigated as a possible target for miR-mediated therapeutics in this kind of cancer. Let-7a is also identified as a direct target for the EWS/FLI1 fusion transcript [77]. The role of miRs as biomarkers in ES has been investigated [90,91]. It has been shown that patients with high levels of miR-34a have better 5-year survival outcomes than patients with low levels of miR-34a [90]. An integrated analysis study of miRs and copy number changes revealed around 20 differentially expressed miRs in chromosomal regions with altered copy numbers. There are around 35 miRs have been shown to have distinct expression patterns in ES tissues and cell lines in comparison with MSCs [92]. Furthermore, the generated results showed that miR-31 act as a tumor suppressor that affected proliferation and invasion in Ewing cell lines.

The expression levels of miR-100, miR-125b, miR-22, miR-221/222, miR-27a, and miR-29a are also suppressed upon suppression of EWS/FLI1 in ES cells. These miRs regulate the insulin-like growth factor (IGF) pathway and promote ES cell growth [12]. Moreover, miR-145 also controls the expression level of EWS/FLI1 and suppresses the ES cell growth [89]. The EWS/FLI1 fusion protein upregulates the EYA3 by suppressing miR-708 and as a result it may be considered as a drug target for the designing of novel chemical scaffolds against ES [93]. Another cell surface glycoprotein CD99 is present in ES tissue, and strongly related to ES malignancy and expression of EWS/FLI1 [94]. Expression of CD99 is regulated by miR-34a via the Notch signalling pathway and prevents ES differentiation. In addition, CD99 is also expressed in the membrane-bound extracellular vesicles, called exosomes derived from ES cells [94]. The analysis of 49 primary ES tumours revealed that miR-34a is related to ES progression and its low expression predicts poorer outcomes and overall worse survival. The IGF-1/AKT/mTOR pathway is also one of the key targets for ES treatment and IGF receptor 1 (IGF-1R) and mTOR inhibitors prevent tumour growth and improve ES survival rate in a mouse xenograft model [95].

However, besides these miRs, there are some other long non-coding RNAs (lncRNAs) that have been shown to affect various aspects of carcinogenesis mostly through modulation of the function of cancer-associated miRs [96]. Deregulation of the miRs is a frequent feature of several illnesses. The development of miR therapies for the treatment of a wide range of human illnesses is therefore the focus of many different sorts of study. The first-ever miRNA therapy is undergoing phase II clinical trials for the treatment of hepatitis C virus (HCV) infection. Miravirsen is a short-locked nucleic acid (LNA) medication targeting miR-122 that is quickly making its way from the lab to the clinic. Novel nucleic acid analogues called LNAs have some therapeutic potential. They are essentially modified or customized RNA nucleotides [97].

miRs have shown potential implications in the treatment of ES, a rare and aggressive form of bone and soft tissue cancer however, current research has explored different promising strategies and applications of miRs in ES therapy [98]. In tumor suppression, multiple miRs function as tumor suppressors by inhibiting the expression of genes involved in cancer growth and metastasis [99]. Researchers have identified miRs that are downregulated in ES and have explored the use of miR mimics to restore their levels. These miRNA mimics can potentially inhibit ES cell growth and induce apoptosis (cell death). Secondly, miRs target and downregulate oncogenes (genes that promote cancer), therefore, modulating the activity of miRs helps to suppress the expression of oncogenes that drive ES development and progression along with metastatic inhibition. Another significant application of miRs is chemotherapy sensitization; miRs modulate the sensitivity of cancer cells to chemotherapy drugs. Researchers have investigated the potential of miRs to sensitize ES cells to chemotherapy, making them more responsive to standard treatments. Most importantly, miR-based therapies also support traditional treatments such as chemotherapy and radiation therapy to achieve synergistic effects. Therefore, combinations of miR inhibitors with other drugs enhance the therapeutic response [100]. Moreover, miR profiling of ES tumors supports theidentification of specific miR expression patterns associated with disease aggressiveness and prognosis. This information can guide treatment decisions and the development of personalized therapeutic strategies against ES. It’s noteworthy that miR-based therapies hold promise for ES treatment, further research is needed to fully understand the mechanisms and optimize their use in clinical settings for ES therapy in the future.

## 6. Conclusions and Future Prospectus

ES causes significant mortality due to the low effectiveness of currently available treatments [101]. Despite the fact that surgical interventions have improved the prognosis, the substantial risk of local relapse makes ES treatment ineffective. Due to the lack of precise information on the molecular pathways involved in ES pathogenesis, the early identification of ES is a significant problem. Therefore, developing new therapeutic techniques for therapy necessitates a deeper understanding of ES and its pathogenic process. There has been a rapid accumulation of evidence over the past ten years supporting the crucial role that unregulated miRs play in the development of human cancers [102,103]. This creates enormous potential for innovative developments in diagnostic and therapeutic strategies in the management of malignant diseases, including ES.

Our knowledge of cancer biology has been significantly improved by the discovery of miRs as post-transcriptional regulators of gene expression, particularly for ES. Although the results are intriguing, more research is required to fully understand the biological functions of miRs and to investigate any potential clinical applications. It is challenging to find consistent miR signatures for the prognosis, diagnosis, and treatment of sarcomas since miR expression is tissue-specific and can be disrupted by a variety of other variables such as infection, hypoxia, pathology, and cytotoxic treatment. miR-directed therapies may have unfavorable side effects because miRs also function in cell signaling networks and affect the expression of several genes. There are multiple miR-based drugs under clinical trials against different diseases which open the new gateways for ES. The miR data could be used as new biomarkers and therapeutic targets to design novel chemical scaffolds for the treatment of ES. Nonetheless, the potential therapeutic utilization of miRs may reshape our understanding of tumorigenesis and will improve the management of ES in the future, potentially giving rise to miR-based medicines and vaccines that could be innovative for paediatric sarcoma therapies, particularly for ES.

## Figures and Tables

**Table 1:** miRs and their possible target genes.

miRs	Target Genes	References
miR-20b	TGFRB2	[45]
miR-130b	CDC42, ARHGAP1	[46]
miR-34b	Notch1	[68]
miR-708-5p	ROCK1-2, MMP-12	[9]
miR-181c	TNSFRSF6	[9]
miR-193b	ErbB4-C	[9,55]
miR-124	SLUG, CCND2	[69]
miR-30d	MMP-2, MMP-9, Bax, Bcl-2	[70]
miR-107	HIF-1β	[61]
miR-185	E2F6	[71]
miR-683	VEGFA	[9]
miR-21	ALCAM	[66]

**Table 2:** Let-7 family in association with carcinogenesis genes.

miRs	Genes	Diseases	Ref.
Let-7a	EWSR1	ES	[77]
	NF2	Meningioma, ES	[72]
	E2F2	Retinoblastoma, Prostate Cancer	[78]
	IL6	ES, Kaposi Sarcoma	[79]
	CCR7	ES	[80,81]
Let-7b	pERK	ES	[82,83]
	EWS-ERG	ES	[82]
	RAS, HIF-1α	ES	[84]
Let-7g	CCND1	ES	[85]
	COL1A2	hepatocarcinoma	[86]

## Data Availability

Not applicable.
